
JOURNAL INFORMATION
==============================
NLM Title Abbreviation: ScientificWorldJournal
Journal ID: ScientificWorldJournal
NLM Journal ID: 101131163
Journal ID: TSWJ

The Scientific World Journal

ISSN: 2356-6140
EISSN: 1537-744X
Publisher: Wiley

ARTICLE INFORMATION
==============================
PMCID: PMC5901211
DOI: 10.1100/tsw.2007.225
Article ID: 380538
Article version: 1
Subjects: Publisher's Note

Publication of a Special Issue: Eicosanoid Receptors and Their Role in Regulating Immune and Inflammatory Responses

Redhead Claire
Publication date: 2007
Electronic publication date: 2007 Aug 9
Volume: 7
First page: 1149
Last page: 1150
Copyright: Copyright © 2007 Claire Redhead.
Copyright year: 2007
License: This is an open access article distributed under the Creative Commons Attribution License, which permits unrestricted use, distribution, and reproduction in any medium, provided the original work is properly cited.
License URL: https://creativecommons.org/licenses/by/3.0/

==============================
Publisher's Note
Special Issue: Eicosanoid Receptors and Inflammation
*TheScientificWorldJOURNAL*, (2007) 7, 1149–1150
ISSN 1537-744X; DOI 10.1100/tsw.2007.225

TheScientificWorldJOURNAL
www.thescientificworld.com

### Publication of a Special Issue: Eicosanoid Receptors and Their Role in Regulating Immune and Inflammatory Responses

Claire Redhead
*TheScientificWorld*

**E-mail:** credhead@thescientificworld.com

*Published August 9, 2007*

---

TheScientificWorldJOURNAL is pleased to have published the special issue 'Eicosanoid Receptors and Their Role in Regulating Immune and Inflammatory Responses', a collection of peer-reviewed papers on a range of key topics in this field. The issue has been organized by Dr. Charles Brink, (CNRS CR-1, Paris, France) with the assistance of Dr. Xavier Norel. Specific topics covered are:

Activation and Regulation of Cellular Eicosanoid Biosynthesis.
**Thomas G. Brock and Marc Peters-Golden**

Structural Manipulation of Eicosanoid Receptors and Cellular Signaling.
**Charles Brink**

Regulation of Immune Cells by Eicosanoid Receptors.
**Nancy D. Kim and Andrew D. Luster**

Prostaglandin Receptor Signaling in Disease.
**Toshiyuki Matsuoka and Shuh Narumiya**

Cytokine-Leukotriene Receptor Interactions.
**Marek Rola-Pleszczynski and Jana Stankova**

Prostanoid Receptors in the Human Vascular Wall.
**Xavier Norel**

Cysteinyl-Leukotriene Receptors and Cellular Signals.
**G. Enrico Rovati and Valerie Capra**

Lipoxin Receptors.
**Mario Romano, Irene Recchia and Antonio Recchiuti**

The Role of Leukotriene Receptor Signaling in Inflammation and Cancer.
**Ramin Massoumi and Anita Sjölander**

©2007 with author.
Published by TheScientificWorld: www.thescientificworld.com

Leukotriene Receptors: Crucial Components in Vascular Inflammation.
**Magnus Bäck**

Endogenous Receptor Agonists: Resolving Inflammation.
**Gerard Bannenberg, Makoto Arita and Charles N. Serhan**

The Publisher wishes to thank the authors and reviewers, and especially acknowledges the contribution of Dr. Brink in bringing about the publication of this special issue.

**This article should be cited as follows:**

Redhead, C. (2007). Publication of a Special Issue: Eicosanoid receptors and their role in regulating immune and inflammatory responses. *TheScientificWorldJOURNAL* **7**, 1149–1150. DOI 10.1100/tsw.2007.225.
